# Nailfold Capillaroscopy in Systemic Sclerosis Patients with and without Pulmonary Arterial Hypertension: A Systematic Review and Meta-Analysis

**DOI:** 10.3390/jcm10071528

**Published:** 2021-04-06

**Authors:** Ioanna Minopoulou, Marieta Theodorakopoulou, Afroditi Boutou, Alexandra Arvanitaki, Georgia Pitsiou, Michael Doumas, Pantelis Sarafidis, Theodoros Dimitroulas

**Affiliations:** 1 Fourth Department of Internal Medicine, Hippokration University Hospital, Medical School, Aristotle University of Thessaloniki, 54642 Thessaloniki, Greece; minopoulouioanna@gmail.com (I.M.); alexandra.arvanit@gmail.com (A.A.); 2 Department of Nephrology, Hippokration University Hospital, Medical School, Aristotle University of Thessaloniki, 54642 Thessaloniki, Greece; marietatheod@gmail.com (M.T.); psarafidis11@yahoo.gr (P.S.); 3 Department of Respiratory Medicine, G. Papanikolaou Hospital, 57010 Pylaia-Chortiatis, Greece; afboutou@yahoo.com; 4 Adult Congenital Heart Centre and National Centre for Pulmonary Arterial Hypertension, Royal Brompton Hospital, Guy’s and St Thomas’ NHS Foundation Trust, Imperial College, London SW3 6NP, UK; 5 Department of Respiratory Failure, G. Papanikolaou Hospital, Aristotle University of Thessaloniki, 57010 Pylaia-Chortiatis, Greece; gpitsiou@yahoo.gr; 6 Second Propedeutic Department of Internal Medicine, Hippokration University Hospital, Medical School, Aristotle University of Thessaloniki, 54642 Thessaloniki, Greece; michalisdoumas@yahoo.co.uk

**Keywords:** pulmonary arterial hypertension, systemic sclerosis, nailfold capillaroscopy, meta-analysis

## Abstract

Systemic sclerosis (SSc)-related pulmonary arterial hypertension (SSc-PAH) is a leading cause of mortality in SSc. The extent of peripheral microvasculopathy assessed through nailfold capillaroscopy might correlate with the presence of PAH in SSc patients. We searched the PubMed, Cochrane Library, Scopus, and Web of Science databases and performed a random effects meta-analysis of observational studies comparing nailfold capillaroscopic alterations in SSc-PAH versus SSc-noPAH patients. Weighted mean differences (WMD) with the corresponding confidence intervals (CIs) were estimated. The quality of the included studies was evaluated using a modified Newcastle–Ottawa scale. Seven studies with 101 SSc-PAH and 277 SSc-noPAH participants were included. Capillary density was marginally reduced in the SSc-PAH group (WMD: −1.0, 95% CI: −2.0 to 0.0, I^2^ = 86%). This effect was strengthened once PAH diagnosis was confirmed by right heart catheterization (WMD: −1.2, 95% CI: −2.3 to −0.1, I^2^ = 85%). An increase in capillary loop width was observed in SSc-PAH compared to SSc-noPAH patients (WMD: 10.9, 95% CI: 2.5 to 19.4, I^2^ = 78%). Furthermore, SSc-PAH patients had a 7.3 times higher likelihood of active or late scleroderma pattern (95% CI: 3.0 to 18.0, I^2^ = 4%). SSc-PAH patients presented with worse nailfold capillaroscopic findings compared to SSc-noPAH patients.

## 1. Introduction

Systemic sclerosis (SSc) is an autoimmune connective tissue disease characterized by microvascular damage and extensive skin and visceral organ fibrosis. These mechanisms synergistically lead to severe internal organ impairment and, subsequently, to increased mortality [1]. Despite better understanding of its pathogenesis, SSc remains a devastating disease with a calculated pooled standardized mortality ratio of 3.5 [2], predominantly due to cardiopulmonary involvement [3]. Pulmonary arterial hypertension related to SSc (SSc-PAH) occurs in approximately 10–15% of SSc individuals and accounts for a significant proportion of early mortality in this population [4,5]. Prompt diagnosis of SSc-PAH and initiation of targeted therapy are of paramount importance [6,7,8], since timely intervention has been associated with improved survival [9].

Nailfold capillaroscopy is a non-invasive diagnostic tool in SSc, allowing clinicians to assess microvascular damage in early stages of the disease [10]. It is included in the latest classification criteria for SSc [11] as well as in the criteria for the very early diagnosis of SSc (VEDOSS) [12]. Recently, nailfold capillaroscopy has also emerged as a potential surrogate marker of SSc progression [13] as several studies have demonstrated that microvascular alterations might correlate with visceral organ involvement, particularly vascular complications such as PAH [13,14,15]. The concept that peripheral microcirculatory changes may be suggestive of a more systemic vascular disorder including pulmonary vasculature is gaining ground [16,17].

Subsequently, the diagnostic accuracy of nailfold videocapillaroscopy [18] as well as its potential role as a screening tool in SSc-PAH have been previously evaluated [19] in systematic reviews, both of which indicated reduced capillary density as a marker of pulmonary vasculopathy. However, no meta-analysis has yet explored the whole spectrum of nailfold capillaroscopic changes in SSc-PAH compared to SSc-noPAH patients. Within this framework, we performed a systematic review and meta-analysis aiming to assess the nailfold capillaroscopic differences between these two SSc populations.

## 2. Materials and Methods

We performed a systematic review and meta-analysis and reported its findings according to the Preferred Reporting Items for Systematic Reviews and Meta-Analyses (PRISMA) statement [20]. We prespecified objectives and methods in a protocol registered on the International Prospective Register of Systematic Reviews PROSPERO (CRD42021235174) and revised some methods in response to peer review comments.

### 2.1. Search Strategy

Two reviewers (IM, MT) systematically searched the PubMed, Web of Science, Scopus, and Cochrane Library databases from inception to February 2021 using a combination of free text and index terms. Reference lists of all eligible articles and relevant reviews were perused. Forward citation searching using Google Scholar was also conducted in order to identify further eligible articles. No language restrictions were posed. Details of the applied search strategy are presented in Data Supplement 1.

### 2.2. Study Selection and Eligibility Criteria

We included observational studies assessing nailfold capillary changes in SSc patients with PAH versus SSc patients without PAH, utilizing any method of nailfold capillaroscopy. PAH was diagnosed based on either right heart catheterization (RHC) or transthoracic echocardiography. Although RHC is the gold-standard method for PAH diagnosis [21], studies utilizing echocardiography were included, since we anticipated a limited number of relevant studies. No restrictions regarding nailfold capillaroscopy equipment were applied. While nailfold videocapillaroscopy (NVC) is the preferred tool for peripheral microcirculation evaluation [22], almost one third of European rheumatology centers utilize a method other than NVC, with nailfold microscopy being the most commonly used after NVC [13]. Any studies that reported comparisons between SSc-PAH and SSc-noPAH were considered eligible. Retrieved publications were imported into reference management software for deduplication. Subsequently, two reviewers (IM, MT) independently screened the titles and abstracts of the identified records. Potentially eligible studies were assessed as full texts. Two disagreements occurred during the study selection process. Both were resolved through consensus.

### 2.3. Data Extraction and Quality Assessment

Data extraction was performed independently by two reviewers (IM, MT) using a predefined Microsoft Excel spreadsheet. From each study, we retrieved information regarding the study and participants’ baseline characteristics as well as nailfold capillaroscopic outcomes. Nailfold capillaroscopic outcomes included quantitative, semi-quantitative, and qualitative parameters. Among the quantitative parameters, information was extracted concerning capillary density, loop width, megacapillaries, combination of dilated and megacapillaries and hemorrhages. Data for bushy capillaries were not extracted, since inter-rater reliability for the assessment of capillary ramifications was considered low [23]. Semi-quantitative parameters included vascular deletion score (avascular areas grade) [24] and nailfold capillaroscopy rating scale score [25,26], while classification of scleroderma pattern as defined by Cutolo et al. constituted the qualitative parameter [27]. For data extraction from full texts, figures, and summary tables, methods recommended by the Cochrane Collaboration were employed.

Two reviewers independently assessed the risk of bias within the selected studies using a modified Newcastle–Ottawa Scale (Data Supplement 2) [28]. Three disagreements between the reviewers occurring during the study quality assessment process were settled by consensus. Risk of bias across studies, using Egger’s statistical test and visual inspection of funnel plot asymmetry, could not be evaluated due to the small number of studies included [29,30].

### 2.4. Data Synthesis and Statistical Analysis

We conducted a meta-analysis when at least two studies provided relevant data for a specific outcome by using an inverse variance random-effects model. Weighted mean differences (WMDs) and 95% confidence intervals (CIs) were calculated for continuous outcomes, whereas odds ratios (ORs) and 95% CIs were calculated for dichotomous outcomes. In particular, capillary density served as our primary outcome, while loop width, megacapillaries, combination of dilated and megacapillaries, hemorrhages, vascular deletion score greater than 1, nailfold capillaroscopy rating scale score greater than 1, and severe pattern defined as active or late scleroderma pattern were our secondary outcomes.

Heterogeneity was assessed using the I^2^ statistic and its significance was determined with the Cochran’s Q test [31]. I^2^ values above 50% suggested substantial heterogeneity [31]. We attempted to explore heterogeneity for our primary outcome with a sensitivity analysis that included only studies of high or moderate quality. In addition, a sensitivity analysis was conducted for our primary outcome including studies providing data for disease duration as capillary density may be affected during long-standing disease [27]. Furthermore, sensitivity analyses were performed for capillary density and capillary loop width excluding studies that utilized (i) transthoracic echocardiography for PAH diagnosis and (ii) nailfold microscopy for peripheral microcirculation evaluation. However, a similar approach could not be applied to the rest of our outcomes; data for severe pattern, vascular deletion score >1, nailfold capillaroscopy rating scale score >1, and hemorrhages were provided only by RHC and NVC studies, whereas the number of studies providing data for megacapillaries and the combination of dilated and megacapillaries was not sufficient for sensitivity analyses. All statistical analyses were performed using the R statistical software (version 3.6.3)

## 3. Results

### 3.1. Search Results, Study Characteristics and Quality Assessment

A total of 220 records were initially retrieved. After removing duplicates, 137 abstracts were evaluated. Of these, 27 abstracts fulfilled our inclusion criteria and remained for full-text assessment. 15 studies were excluded for various reasons (Data Supplement 3). Ultimately, 12 studies were included in the qualitative synthesis [25,32,33,34,35,36,37,38,39,40,41,42], and seven studies were included in the quantitative synthesis of our study [25,35,36,37,38,39,40]. The step-by-step study selection process is illustrated in Data Supplement 4. Among the studies included, 10 were of a cross-sectional design [25,32,33,34,35,36,37,38,39,40], while two were longitudinal [41,42].

A total of 702 SSc patients were included in this study. Among them, 147 individuals (20.9%) were diagnosed with SSc-PAH. Mean age of the SSc-PAH group was 54.0 ± 14.0 years, while mean disease duration was 15.9 ± 14.4 years. Accordingly, in the SSc-noPAH group, mean age was 52.8 ± 14.0 years, whereas disease duration was 12.9 ± 10.3 years. PAH was diagnosed by RHC in 7 studies [25,35,36,37,40], while transthoracic echocardiography or a combination of RHC and echocardiography were used for PAH diagnosis in four studies [33,34,38,39]. In one study, the method of PAH diagnosis was not defined [32]. NVC was used as an evaluation method in eight studies [25,32,35,36,37,40], while nailfold microscopy was utilized in four studies [33,34,38,39]. Only five studies provided data for concomitant use of vasodilatory treatment [34,35,36,41,42]. Of note, Hofstee et al. found no differences between treated and not treated patients with SSc-PAH regarding capillary density [35], whereas Avouac et al. reported no significant impact of baseline vasodilator treatment on the progression of microhemorrhages, giant capillaries, capillary loss, and neoangiogenesis [42]. The characteristics of all included studies are depicted in Table 1.

Applying our modified Newcastle–Ottawa scale, four studies were considered of good [25,35,41,42] and three studies of moderate quality [36,37,38], whereas five studies were deemed of poor quality [32,33,34,39,40] (Data Supplement 5).

### 3.2. Quantitative Assessment

**Figure 1:**
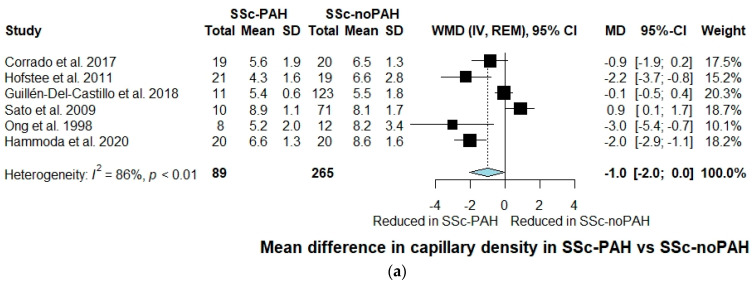

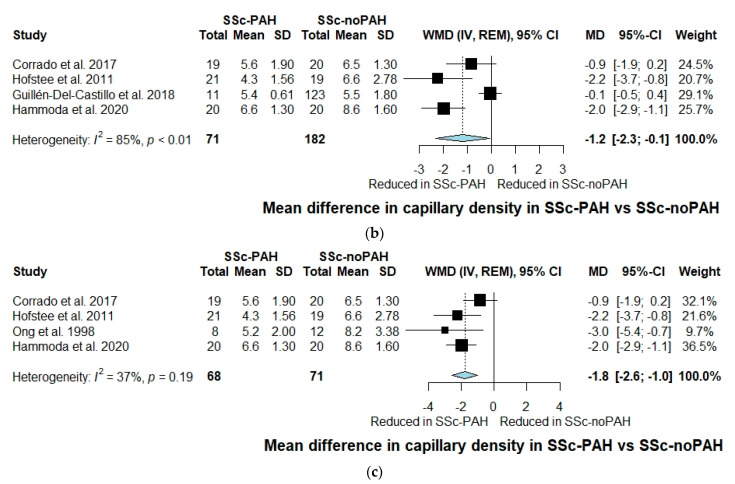
(**a**) Forest plot of observational studies exploring capillary density in SSc-PAH versus SSc-noPAH patients. CI: confidence interval; IV: inverse variance; MD: mean difference; REM: random effects model; SD: standard deviation; SSc-noPAH: Systemic sclerosis without \pulmonary arterial hypertension; SSc-PAH: Systemic sclerosis with pulmonary arterial hypertension; WMD: weighted mean difference. (**b**) Sensitivity analysis including only studies utilizing right heart catheterization as a method of SSc-PAH diagnosis; forest plot of observational studies exploring capillary density in SSc-PAH versus SSc-noPAH patients. CI: confidence interval; IV: inverse variance; MD: mean difference; REM: random effects model; SD: standard deviation; SSc-noPAH: Systemic sclerosis without pulmonary arterial hypertension; SSc-PAH: Systemic sclerosis with pulmonary arterial hypertension; WMD: weighted mean difference. (**c**) Sensitivity analysis including only studies of good and moderate quality; forest plot of observational studies exploring capillary density in SSc-PAH versus SSc-noPAH patients. CI: confidence interval; IV: inverse variance; MD: mean difference; REM: random effects model; SD: standard deviation; SSc-noPAH: Systemic sclerosis without pulmonary arterial hypertension; SSc-PAH: Systemic sclerosis with pulmonary arterial hypertension; WMD: weighted mean difference.

Overall, six studies with 354 participants assessed capillary density during nailfold capillaroscopy evaluation [35,36,37,38,39,40]. Capillary density was reduced in SSc-PAH compared to SSc-noPAH patients, without, however, reaching statistical significance (WMD: −1.0, 95% CI: −2.0 to 0.0, I^2^ = 86%) (Figure 1a). Nevertheless, when a sensitivity analysis was performed including only studies that utilized RHC for PAH diagnosis, capillary density was significantly reduced in the SSc-PAH group (WMD: −1.2, 95% CI: −2.3 to −0.1, I^2^ = 85%) (Figure 1b). When excluding studies that assessed peripheral microcirculation by nailfold microscopy, capillary density was again found significantly reduced in SSc-PAH patients (WMD: −1.2, 95% CI: −2.3 to −0.1, I^2^ = 85%) (Data Supplement 6; Figure 6.1). Similarly, when sensitivity analyses were conducted excluding studies of low quality and studies that did not provide data for disease duration, a further reduction of capillary density in the SSc-PAH group was observed ((WMD: −1.8, 95% CI: −2.6 to −1.0, I^2^ = 37%) (Figure 1c) and (WMD: −1.8, 95% CI: −2.6 to −1.0, I^2^ = 37%)) (Data Supplement 6; Figure 6.2), respectively).

**Figure 2:**
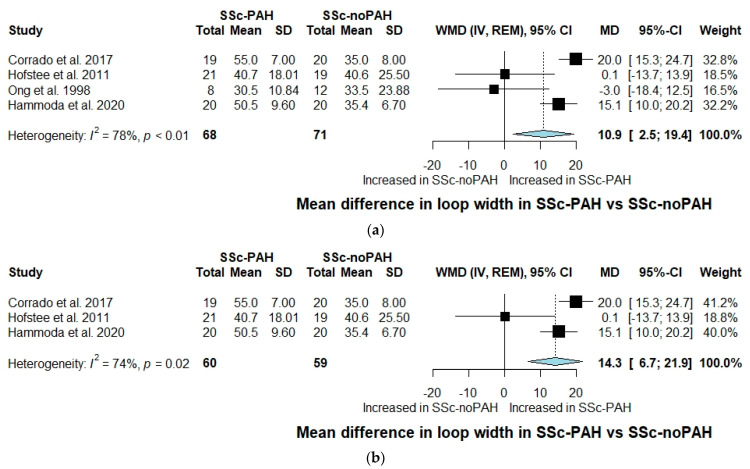
(**a**) Forest plot of observational studies exploring capillary loop width in SSc-PAH versus SSc-noPAH patients. CI: confidence interval; IV: inverse variance; MD: mean difference; REM: random effects model; SD: standard deviation; SSc-noPAH: Systemic sclerosis without pulmonary arterial hypertension; SSc-PAH: Systemic sclerosis with pulmonary arterial hypertension; WMD: weighted mean difference. (**b**) Sensitivity analysis including only studies utilizing right heart catheterization for SSc-PAH diagnosis; forest plot of observational studies exploring capillary loop width in SSc-PAH versus SSc-noPAH patients. CI: confidence interval; IV: inverse variance; MD: mean difference; REM: random effects model; SD: standard deviation; SSc-noPAH: Systemic sclerosis without pulmonary arterial hypertension; SSc-PAH: Systemic sclerosis with pulmonary arterial hypertension; WMD: weighted mean difference.

With regard to capillary loop width, a significant increase was observed in patients with SSc-PAH compared to SSc-noPAH patients (WMD: 10.9, 95% CI: 2.5 to 19.4, I^2^ = 78%) (Figure 2a). A further increase of capillary loop width in the SSc-PAH group was noted when performing a sensitivity analysis including studies utilizing only RHC as a method of PAH diagnosis (WMD: 14.3, 95% CI: 6.7 to 21.9, I^2^ = 74%) (Figure 2b). Accordingly, when conducting a sensitivity analysis including only studies utilizing NVC, capillary loop width was significantly increased in SSc-PAH compared to SSc-noPAH patients (WMD: 14.3, 95% CI: 6.7 to 21.9, I^2^ = 74%) (Data Supplement 6; Figure 6.3).

No statistically significant differences concerning megacapillaries or the combination of dilated and megacapillaries were observed between the two groups (WMD: 0.9, 95% CI: −1.4 to 3.3, I^2^ = 100% and WMD: −0.9, 95% CI: −2.4 to 0.5, I^2^ = 78%, respectively) (Data Supplement 6; Figure 6.4 and Figure 6.5, respectively). Similarly, no statistical differences for the odds of hemorrhages were detected between the two groups (OR: 3.4, 95% CI: 0.4 to 30.2, I^2^ = 74%) (Data Supplement 6; Figure 6.6).

### 3.3. Semi-Quantitative and Qualitative Assessment

The odds for vascular deletion score greater than 1 were higher among patients with SSc–PAH compared to patients without PAH (OR: 30.9, 95% CI: 7.7 to 124.2, I^2^ = 0%) (Data Supplement 7; Figure 7.1). Similarly, nailfold capillaroscopy rating scale score above 1 was more frequent in the SSc-PAH group (OR: 30.4, 95% CI: 6.0 to 154.5, I^2^ = 0%) (Data Supplement 7; Figure 7.2).

**Figure 3:**
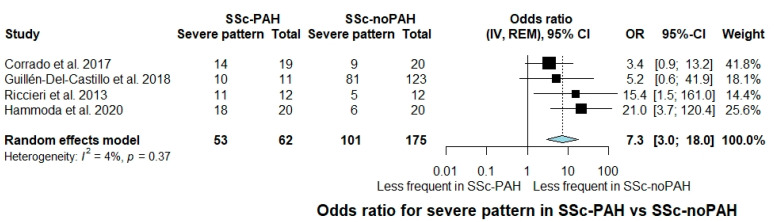
Forest plot of observational studies exploring occurrence of severe pattern (active/late) in SSc-PAH versus SSc-noPAH patients. CI: confidence interval; IV: inverse variance; SSc-PAH: Systemic sclerosis with pulmonary arterial hypertension; SSc-noPAH: Systemic sclerosis without pulmonary arterial hypertension; MD: mean difference; SD: standard deviation; WMD: weighted mean difference; REM: random effects model.

Severe pattern was evaluated across four studies [25,36,37,40]. The OR for the presence of severe pattern in SSc-PAH individuals was 7.3 compared to SSc-noPAH individuals (95% CI: 3.0 to 18.0, I^2^ = 4%) (Figure 3).

## 4. Discussion

In this systematic review and meta-analysis, we explored the differences in nailfold capillaroscopic alterations between SSc-PAH and SSc-noPAH individuals. We demonstrated that capillary density was reduced in SSc-PAH patients and this effect was accentuated when RHC was utilized for PAH diagnosis. Accordingly, SSc-PAH individuals had more than thirty times higher likelihood of vascular deletion score greater than 1. Increased capillary loop width, severe scleroderma pattern, and nailfold capillaroscopy rating scale score greater than 1 were also associated with the existence of PAH. The results of our analysis indicate that despite overall microvascular injury in SSc patients, those with SSc-PAH present with more advanced changes in peripheral microcirculation.

Microvasculopathy represents a fundamental part of SSc pathogenesis, leading to several clinical manifestations such as Raynaud’s phenomenon and digital ulcers [45]. Multiple cohort studies have postulated that nailfold capillaroscopic abnormalities correlate with the severity of organ involvement including PAH [13,41,42,46]. Indeed, in a 3-year longitudinal study, progressive loss of capillaries, presence of angiogenesis, and late scleroderma pattern were identified as markers for the occurrence of PAH [42]. SSc-PAH has also been associated with higher scores for capillary loss and capillary disorganization [41]. Additionally, it has been reported that increasing echocardiographically derived systolic pulmonary arterial pressure correlates with the severity of scleroderma pattern [46]. Furthermore, a diagnostic accuracy meta-analysis assessing the value of NVC in the diagnosis of SSc-PAH demonstrated that lower capillary density and increased capillary loop width could potentially detect patients with SSc-PAH [18]. These findings are consistent with the results of our study, suggesting that a higher degree of peripheral nailfold microangiopathy is more common in SSc-PAH, supporting the hypothesis that peripheral microvascular changes may parallel with similar abnormalities in the pulmonary vascular tree.

Interestingly, changes in peripheral microcirculation have also been observed in other forms of pulmonary hypertension [35,36,47,48,49,50]. Hofstee et al. suggested that capillary density is significantly reduced in individuals with idiopathic PAH compared to healthy controls and inversely correlated with the severity of idiopathic PAH [35]. These findings have later been confirmed by other studies, which additionally reported a significant increase in capillary loop width in idiopathic PAH patients [36,47]. Similar patterns of capillaroscopic abnormalities have been reported in chronic thromboembolic pulmonary hypertension [47] and in connective tissue disease-related PAH beyond SSc [49]. For example, the incidence of scleroderma pattern as well as high vascular deletion score were established as independent predictors of PAH in systemic lupus erythematous patients [49]. Recently, a cross-sectional study showed that patients with Eisenmenger syndrome had a reduced capillary density, an increased loop width and more abnormal capillaries than age- and sex-matched healthy controls. NVC shape abnormalities in Eisenmenger syndrome were positively correlated with N-terminal-pro brain natriuretic peptide and negatively associated with estimated glomerular filtration rate [50]. The presence of reduced capillary density and increased capillary width across the whole spectrum of pre-capillary PAH might indicate that besides SSc-associated microangiopathy, PAH may serve as an additional contributing factor to the pronounced microvascular abnormalities observed in SSc-PAH compared to SSc-noPAH.

Early detection of SSc-PAH remained an unmet need for years. According to recent studies from European PAH registries, the percentage of SSc-PAH presenting with New York Heart Association (NYHA) functional class III or IV symptoms at the time of diagnosis reaches 75% [51,52], and remains approximately as high as 30 years ago [53]. Recently, significant progress has been marked with the development of risk assessment algorithms such as the DETECT and Australian Scleroderma Interest Group (ASIG) algorithm as well as the latest European Society of Cardiology (ESC)/European Respiration Society (ERS) guidelines [21,54,55]. Studies have demonstrated that the implementation of these algorithms annually in asymptomatic patients reduces or even eliminates missed PAH cases [56,57,58]. However, the high referral rate for RHC as well as the unclear cost-effectiveness of yearly screening in asymptomatic patients constitute major throwbacks, leading clinicians to poor adherence to PAH screening guidelines [55,59]. Since nailfold capillaroscopy seems to correlate with the presence of PAH, it could potentially navigate clinicians in PAH risk stratification and enhance the performance characteristics of current algorithms, whilst reducing the rate of needless RHCs.

### 4.1. Strengths and Limitations

To our knowledge, this systematic review and meta-analysis provides the most up-to-date and comprehensive analysis of the effect of PAH on the peripheral microcirculation in SSc patients. Previous secondary studies have evaluated the use of nailfold capillaroscopy in SSc-PAH [18,19]. However, our study is the first to provide a direct comparison between SSc-PAH and SSc-noPAH individuals, demonstrating that SSc-PAH is characterized by progressing peripheral microvascular changes compared to SSc-noPAH. All available methods of nailfold capillaroscopy were included, in order to perform a more inclusive systematic review that might be of interest to clinicians and future researchers, irrespective of the nailfold capillaroscopy equipment they use in their everyday practice. Additionally, we analyzed all nailfold capillaroscopy assessment parameters, using standardized definitions, in order to pinpoint the exact parameters that might be able to discern SSc-PAH from SSc-noPAH individuals and therefore provide meticulous directions for future research. As a result, capillary density was not the only capillaroscopic parameter linked with SSc-PAH, but we further expanded previous findings by establishing increased capillary loop width and severe scleroderma pattern as markers of SSc-PAH. Given the scarcity of available evidence resulting from the rarity of the disease itself, our meta-analysis, on top of previous studies [18,19], further reinforces the hypothesis that nailfold capillaroscopy might be a useful tool for PAH risk stratification in current rheumatology clinical practice.

However, our findings should be interpreted in the context of several limitations relevant to the high risk of bias, significant heterogeneity, and small number of participants among the included studies. Indeed, most studies followed different protocols in terms of PAH and SSc diagnosis as well as nailfold capillaroscopy method. Additionally, absence of matching based on age, disease duration, or concomitant digital ulcers, and lack of blinding of investigators to clinical diagnosis during capillaroscopy raised further methodological concerns. Limitations at the review level were related to the high degree of statistical heterogeneity observed in analyses of capillary density, capillary loop width and dilated and megacapillaries. In an attempt to explore the sources of heterogeneity regarding capillary density as well as capillary loop width, we conducted sensitivity analyses by excluding transthoracic echocardiography, nailfold microscopy, and studies at high risk of bias. Nevertheless, this approach could not be applied to the rest of our outcomes due to the paucity of available data. Furthermore, most included studies did not provide information on concomitant vasodilator medication or clinical data that are associated with the presence of SSc-PAH such as teleangiectasias or anti-centromere antibodies, in a form that would allow us to perform further analyses. Therefore, we were not able to investigate their potential effects on possible differences in nailfold capillaroscopy abnormalities between the groups compared.

### 4.2. Future Perspectives and Conclusions

The results of our analysis suggest that SSc-PAH individuals present with worsening stages of peripheral microangiopathy compared to SSc-noPAH patients, suggesting a more widespread microvasculopathy in these individuals. Future research should explore whether capillaroscopic characteristics are able to identify patients at high risk for developing PAH. Large, well-designed, multi-center, adequately powered RHC-based studies, encompassing sequential nailfold capillaroscopy for longitudinal evaluation of microcirculation, are needed to establish nailfold capillaroscopy as a reliable indicator of pulmonary vasculopathy and determine its performance as part of current screening algorithms in patients at higher risk for SSc-PAH. Such approaches require prespecified methodology for the evaluation of certain capillaroscopic parameters and the application of a homogeneous nailfold capillaroscopy scoring system in order to ensure the validity of measurements [22]. Toward this direction, new automated systems for the calculation of capillary density in NVC images have been introduced with promising results in terms of reliability and time consumption [60]. A potential validation of these findings would showcase the value of nailfold capillaroscopy, particularly NVC, due to the shorter training duration it requires and the better image quality it offers [22], as a useful adjunct for PAH screening and a helpful guide for clinical decision making.

## Figures and Tables

**Table 1:** Characteristics of included studies. ACR: American College of Rheumatology; EULAR: European League against Rheumatism; N/A: Not available; NFC: Nailfold capillaroscopy rating scale score; NVC: Nailfold video-capillaroscopy; PAH: Pulmonary arterial hypertension; RHC: Right heart catheterization; SSc: Systemic Sclerosis; SSc-noPAH: Systemic sclerosis without pulmonary arterial hypertension; SSc-PAH: Systemic sclerosis with Pulmonary arterial hypertension; TTE: Transthoracic echocardiography; VDS: Vascular deletion score.

Study	Study Design	SSc Criteria	Population		Mean Disease Duration (Years)		Method of PAH Diagnosis	Nailfold Capillaroscopy Method	Capillaroscopy Parameters
			SSc- PAH	SSc-noPAH	SSc- PAH	SSc-noPAH			
**Corrado et al.** **2017** [36]	Cross-sectional	ACR/EULAR 2013 diagnostic criteria for SSc [11]	19	20	15.3 ± 3.9	18.4 ± 4.1	RHC	NVC, 200× magnification	Capillary density Capillary dimensions Neoangiogenesis Pattern
**Hofstee et al.** **2009** [35]	Cross-sectional	preliminary criteria for the classification of SSc 1980 [43]	21	19	3.7 ± 12.3	3.7 ± 9.8	RHC	computer-based panorama mosaic NVC	Capillary density Capillary dimensions
**Guillén-Del-Castillo et al. 2018** [40]	Cross-sectional	ACR/EULAR 2013 diagnostic criteria for SSc [11]	11	123	N/A		RHC	NVC, 200× magnification	Capillary density Capillary dimensions Microhemorrhages Neoangiogenesis Pattern
**Sato et al.** **2009** [39]	Cross-sectional	preliminary criteria for the classification of SSc 1980 [43]	10	71	N/A		TTE	microscopy 10–20× magnification	Capillary density Capillary dimensions VDS
**Ong et al.** **1998** [38]	Cross-sectional	Not defined	8	12	22.9 ± 16.6	18.1 ± 10.2	TTE and/or RHC	microscopy 60× magnification	Capillary density Capillary dimensions
**Riccieri et al.** **2013** [25]	Cross-sectional	preliminary criteria for the classification of SSc 1980 [43]	12	12	24.4 ± 17.6	18.9 ± 10.7	RHC	NVC, 200× magnification	Pattern VDS NFC
**Hammoda et al.** **2020** [37]	Cross-sectional	ACR/EULAR 2013 diagnostic criteria for SSc [11]	20	20	21.4 ± 10.4	9.3 ± 6.5	RHC	NVC, 200× magnification	Capillary density Capillary dimensions VDS Microhemorrhages Pattern NFC
**Meier et al.** **2012** [32]	Cross-sectional	Not defined	10	37	N/A		Not defined	NVC	Capillary density Capillary dimensions
**Bredemeier et al.** **2004** [33]	Cross-sectional	preliminary criteria for the classification of SSc 1980 [43]	11	75	N/A		TTE	microscopy 6.5–65× magnification	Capillary dimensions VDS Microhemorrhages
**Greidinger et al.** **2001** [34]	Cross-sectional	Not defined	8	7	N/A		TTE and/or RHC	microscopy	Capillary density Neoangiogenesis
**Avouac et al. 2017** [42]	Longitudinal	ACR/EULAR 2013 diagnostic criteria for SSc [11]	8	132	9 ± 8	9 ± 8	RHC	NVC, 200× magnification	Capillary density Capillary dimensions Microhemorrhages Neoangiogenesis
**Sulli et al. 2012** [41]	Longitudinal	preliminary criteria for the classification of SSc 1980 [43] or LeRoy criteria for the classification of early SSc [44]	4	34	N/A		RHC	NVC, 200× magnification	Capillary dimensions Microhemorrhages Capillary ramifications Pattern NFC

## Supplementary Materials

The following are available online at https://www.mdpi.com/article/10.3390/jcm10071528/s1, Data Supplement 1: PubMed search syntax and search string; Data Supplement 2: Modified Newcastle—Ottawa Scale; Data Supplement 3: Reference list of all excluded studies with reason for exclusion; Data Supplement 4: Flow diagram of study selection process; Data Supplement 5: Risk of bias of observational studies; Data Supplement 6: Forest plots of quantitative parameters of nailfold capillaroscopy assessment; Data Supplement 7: Forest plots of semi-quantitative parameters of nailfold capillaroscopy assessment.
