# Supplementary material for: Nailfold Capillaroscopy in Systemic Sclerosis Patients with and without Pulmonary Arterial Hypertension: A Systematic Review and Meta-Analysis
Source: J Clin Med. 2021 Apr 6;10(7):1528. doi: 10.3390/jcm10071528 (PMC8038744; doi:10.3390/jcm10071528)
Supplement: Supplementary file 1 [file jcm-10-01528-s001.pdf]

Table of Contents

Data Supplement 1: PubMed search syntax and search string **..** ***2***

Data Supplement 2: Modified Newcastle – Ottawa Scale **..** ***3***

Data Supplement 3: Reference list of all excluded studies with reason for exclusion **..........** ***5***

Data Supplement 4: Flow diagram of study selection process **..** ***7***

Data Supplement 5: Risk of bias of observational studies **..** ***8***

Data Supplement 6: Forest plots of quantitative parameters of nailfold capillaroscopy assessment **...** ***9***

Data Supplement 7: Forest plots of semi-quantitative parameters of nailfold capillaroscopy assessment **...** ***15***

Data Supplement 1: PubMed search syntax and search string

Search syntax

ID	Search
#1	pulmonary hypertension [All fields]
#2	pulmonary hypertension [MeSH terms]
#3	#1 OR #2
#4	systemic sclerosis [All fields]
#5	scleroderma [All fields]
#6	scleroderma, systemic [MeSH terms]
#7	#4 OR #5 OR #6
#8	nailfold capillaroscopy [All fields]
#9	nailfold videocapillaroscopy [All fields]
#10	#8 OR #9
#11	#3 AND #7 AND #10

Search string

(((nailfold capillaroscopy) OR (nailfold videocapillaroscopy)) AND ((pulmonary hypertension) OR (pulmonary hypertension[MeSH Terms]))) AND (((systemic sclerosis) OR (scleroderma)) OR (systemic sclerosis[MeSH Terms]))

The search strategy was modified accordingly for the other databases.

Data Supplement 2: Modified Newcastle – Ottawa Scale

Selection: (Maximum 5 stars)

1) Representativeness of the sample:

a) Truly representative of the average in the target population. * (all subjects or random sampling e.g. consecutive patients)

b) Somewhat representative of the average in the target population. * (nonrandom sampling)

c) Selected group of users.

d) No description of the sampling strategy.

2) Sample size:

a) Justified and satisfactory (>30 patients suffering from SSc). *

b) Not justified.

3) Non-respondents:

a) Comparability between respondents and non-respondents characteristics is established, and the response rate is satisfactory. *

b) The response rate is unsatisfactory, or the comparability between respondents and non-respondents is unsatisfactory.

c) No description of the response rate or the characteristics of the responders and the non-responders.

4) Ascertainment of the exposure (pulmonary hypertension):

a) Right heart catheterization **

b) Transthoracic echocardiography *

c) No description of the measurement tool.

Comparability: (Maximum 2 stars)

1) The subjects in different outcome groups are comparable, based on the study design or analysis. Confounding factors are controlled.

a) The study controls for the most important factor (disease duration). *

b) The study control for any additional factor (e.g. digital ulcers, age). *

Outcome: (Maximum 3 stars)

1) Assessment of the outcome:

a) Nailfold capillaroscopy performed by researchers blind to diagnosis. **

b) Nailfold capillaroscopy performed by researchers not blind to diagnosis. *

c) Record linkage.

d) No description.

2) Statistical test:

a) The statistical test used to analyze the data is clearly described and appropriate, and the measurement of the association is presented, including confidence intervals and the probability level (p value). *

b) The statistical test is not appropriate, not described or incomplete.

Good quality: 5 or 4 stars in selection domain AND 2 stars in comparability domain AND 2 or 3 stars in outcome/exposure domain

Moderate quality: 3 or 2 stars in selection domain AND 1 star in comparability domain AND 2 or 3 stars in outcome/exposure domain

Poor quality: 0 or 1 star in selection domain OR 0 stars in comparability domain OR 0 or 1 stars in outcome/exposure domain

Data Supplement 3: Reference list of all excluded studies with reason for exclusion

<u>Data presented in part and then in whole in future publications</u>

1. Bredemeier M, Xavier RM, Capobianco KG, Restelli VG, Rohde LEP, Pinotti AFF, et al. Capilaroscopia periungueal pode sugerir atividade de doença pulmonar na esclerose sistêmica. Rev Bras Reumatol. 2004 Feb;44(1):19–30.

<u>No relevant outcome</u>

1. Voilliot D, Magne J, Dulgheru R, Kou S, Henri C, Caballero L, et al. Prediction of new onset of resting pulmonary arterial hypertension in systemic sclerosis. Arch Cardiovasc Dis. 2016 Apr;109(4):268–77.

2. Marino Claverie L, Knobel E, Takashima L, Techera L, Oliver M, Gonzalez P, et al. Organ involvement in Argentinian systemic sclerosis patients with "late" pattern as compared to patients with "early/active" pattern by nailfold capillaroscopy. Clin Rheumatol. 2013 Jun;32(6):839–43.

3. van Roon AM, Huisman CC, van Roon AM, Zhang D, Stel AJ, Smit AJ, et al. Abnormal Nailfold Capillaroscopy Is Common in Patients with Connective Tissue Disease and Associated with Abnormal Pulmonary Function Tests. J Rheumatol. 2019 Sep;46(9):1109–16.

4. Pizzorni C, Sulli A, Paolino S, Ruaro B, Smith V, Trombetta AC, et al. Progression of Organ Involvement in Systemic Sclerosis Patients with Persistent "Late" Nailfold Capillaroscopic Pattern of Microangiopathy: A Prospective Study. J Rheumatol. 2017 Dec;44(12):1941–2.

5. Shenavandeh S, Haghighi MY, Nazarinia MA. Nailfold digital capillaroscopic findings in patients with diffuse and limited cutaneous systemic sclerosis. Reumatologia. 2017;55(1):15–23.

6. Markusse IM, Meijs J, de Boer B, Bakker JA, Schippers HPC, Schouffoer AA, et al. Predicting cardiopulmonary involvement in patients with systemic sclerosis: complementary value of nailfold videocapillaroscopy patterns and disease-specific autoantibodies. Rheumatol Oxf Engl. 2017 Jul 1;56(7):1081–8.

7. Bournia VK, Kottas K, Panopoulos S, Konstantonis G, Iliopoulos A, Tektonidou MG, et al. Differential performance of nailfold video capillaroscopic parameters in the diagnosis and prognosis of systemic sclerosis. Clin Exp Rheumatol. 2020 Jun;38 Suppl 125(3):29–39.

<u>No full-text available</u>

1. Klyaus NA, Simakova MA, Maslyansky AL, Moiseeva OM. Search for clinical predictors of pulmonary hypertension in patients with systemic sclerosis. Nauchno-Prakticheskaya Revmatologiya. 2018 Jan;56(5):586-90

No data available for systemic sclerosis patients without pulmonary arterial hypertension

1. van Leeuwen NM, Wortel CM, Fehres CM, Bakker JA, Scherer HU, Toes REM, et al. Association Between Centromere- and Topoisomerase-specific Immune Responses and the Degree of Microangiopathy in Systemic Sclerosis. J Rheumatol. 2020 Jun 1;

2. Castellví I, Simeón-Aznar CP, Sarmiento M, Fortuna A, Mayos M, Geli C, et al. Association between nailfold capillaroscopy findings and pulmonary function tests in patients with systemic sclerosis. J Rheumatol. 2015 Feb;42(2):222–7.

3. Kim H-S, Park M-K, Kim H-Y, Park S-H. Capillary dimension measured by computer-based digitalized image correlated with plasma endothelin-1 levels in patients with systemic sclerosis. Clin Rheumatol. 2010 Mar;29(3):247–54.

4. Ingegnoli F, Ardoino I, Boracchi P, Cutolo M, EUSTAR co-authors. Nailfold capillaroscopy in systemic sclerosis: data from the EULAR scleroderma trials and research (EUSTAR) database. Microvasc Res. 2013 Sep;89:122–8.

5. Caraba A, Romosan I, Borza C, Muntean D, Noveanu L. A nailfold capillaroscopy study in patients with systemic sclerosis and pulmonary hypertension. In: Dumitrascu DL, Portincasa P, editors. Proceedings of 49th annual scientific meeting of the European society for clinical investigation. 2015 May 27-30; Cluj-Napoca, Romania. Bologna: MEDIMOND; 2015.p. 83-8

Data for SSc-PAH patients pooled with other population

1. Trombetta AC, Pizzorni C, Ruaro B, Paolino S, Sulli A, Smith V, et al. Effects of Longterm Treatment with Bosentan and Iloprost on Nailfold Absolute Capillary Number, Fingertip Blood Perfusion, and Clinical Status in Systemic Sclerosis. J Rheumatol. 2016 Nov;43(11):2033–41.

Data Supplement 4: Flow diagram of study selection process

Identification

Records identified through database search (n = 215)

- PubMed: 54
- Central: 2
- Scopus: 82
- Web of Science: 77

Additional records identified through forward citation tracking, reference lists of relevant reviews
(n = 5)

Screening

Records after duplicate removal
(n = 137)

Records screened
(n = 137)

Records excluded
(n = 110)

Eligibility

Full-text articles assessed for eligibility
(n = 27)

Full-text articles excluded
(n = 15)

- No data for SSc-noPAH (n = 5)
- Data for SSc-PAH pooled with other population (n = 1)
- Full-text not available (n = 1)
- No relevant outcomes (n = 7)
- Overlapping (n = 1)

Included

Studies included in the qualitative synthesis
(n = 12)

Studies excluded from meta-analysis
(n =5)

- No sufficient data on nailfold capillaroscopy outcomes (n=5)

Studies included in the quantitative synthesis
(n = 7)

Data Supplement 4: Flow diagram of study selection process. SSc-PAH: Systemic sclerosis with Pulmonary arterial hypertension; SSc-noPAH: Systemic sclerosis without Pulmonary arterial hypertension.

Data Supplement 5: Risk of bias of observational studies

Study	Selection	Comparability	Outcome	Overall quality
Corrado et al. 2017	*****	*	***	moderate
Hofstee et al. 2011	*****	**	***	good
Guillén-Del-Castillo et al. 2018	****	-	***	poor
Sato et al. 2009	***	-	***	poor
Ong et al. 1998	**	**	**	moderate
Riccieri et al. 2013	****	**	**	good
Hammoda et al. 2020	****	*	**	moderate
Meier et al. 2012	*	-	*	poor
Bredemeier et al. 2004	**	-	***	poor
Greidinger et al. 2001	**	-	***	poor
Avouac et al. 2017	****	**	***	good
Sulli et al. 2012	****	**	***	good

Data Supplement 5: Within studies risk of bias assessment according to modified Newcastle-Ottawa scale.

Data Supplement 6: Forest plots of quantitative parameters of nailfold capillaroscopy assessment

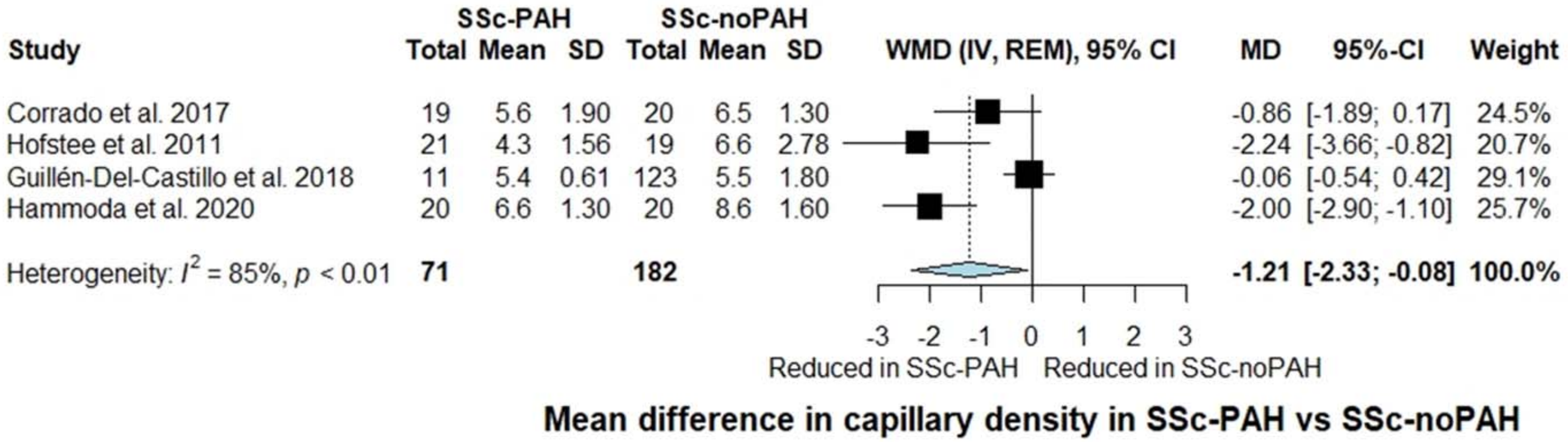

Figure 6. 1: Sensitivity analysis including only studies utilizing nailfold videocapillaroscopy for peripheral microcirculation evaluation; Forest plot of observational studies exploring capillary density in SSc-PAH versus SSc-noPAH patients. CI: confidence interval; IV: inverse variance; MD: mean difference; REM: Random Effects Model; SD: standard deviation; SSc-noPAH: Systemic sclerosis without Pulmonary arterial hypertension; SSc-PAH: Systemic sclerosis with Pulmonary arterial hypertension; WMD: weighted mean difference.

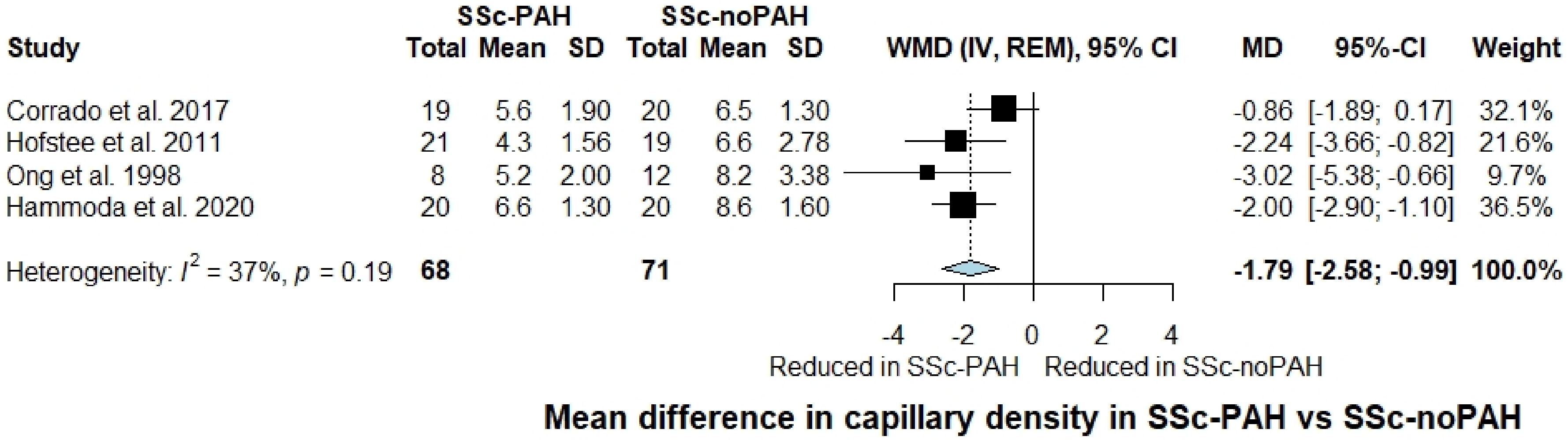

Figure 6.2: Sensitivity analysis including only studies providing data for disease duration; Forest plot of observational studies exploring capillary density in SSc-PAH versus SSc-noPAH patients. CI: confidence interval; IV: inverse variance; MD: mean difference; REM: Random Effects Model; SD: standard deviation; SSc-noPAH: Systemic sclerosis without Pulmonary arterial hypertension; SSc-PAH: Systemic sclerosis with Pulmonary arterial hypertension; WMD: weighted mean difference.

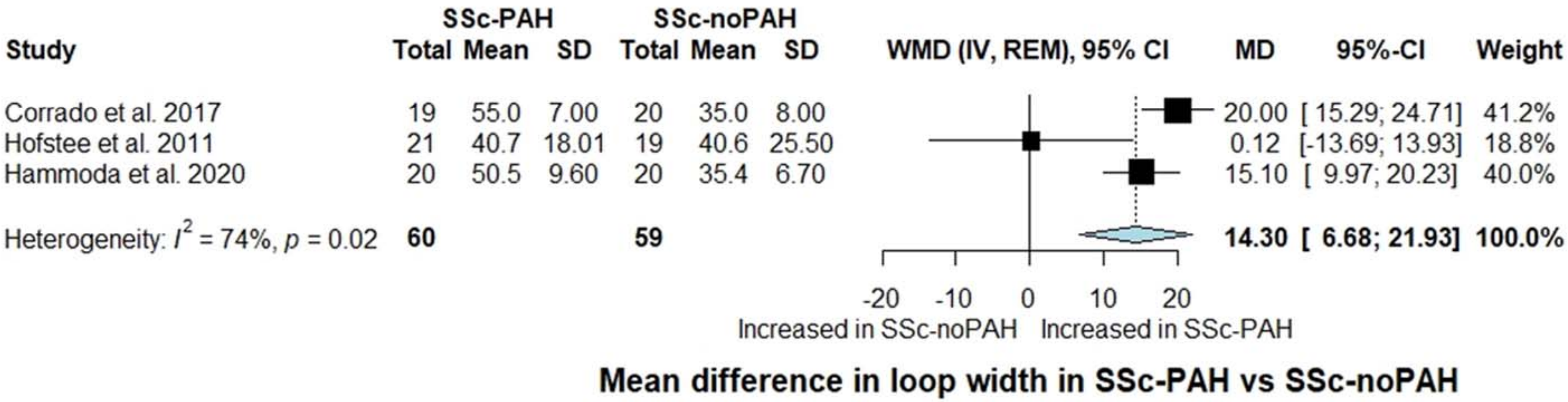

Figure 6.3: Sensitivity analysis including only studies utilizing nailfold videocapillaroscopy for peripheral microcirculation evaluation; Forest plot of observational studies exploring capillary loop width in SSc-PAH versus SSc-noPAH patients. CI: confidence interval; IV: inverse variance; MD: mean difference; REM: Random Effects Model; SD: standard deviation; SSc-noPAH: Systemic sclerosis without Pulmonary arterial hypertension; SSc-PAH: Systemic sclerosis with Pulmonary arterial hypertension; WMD: weighted mean difference.

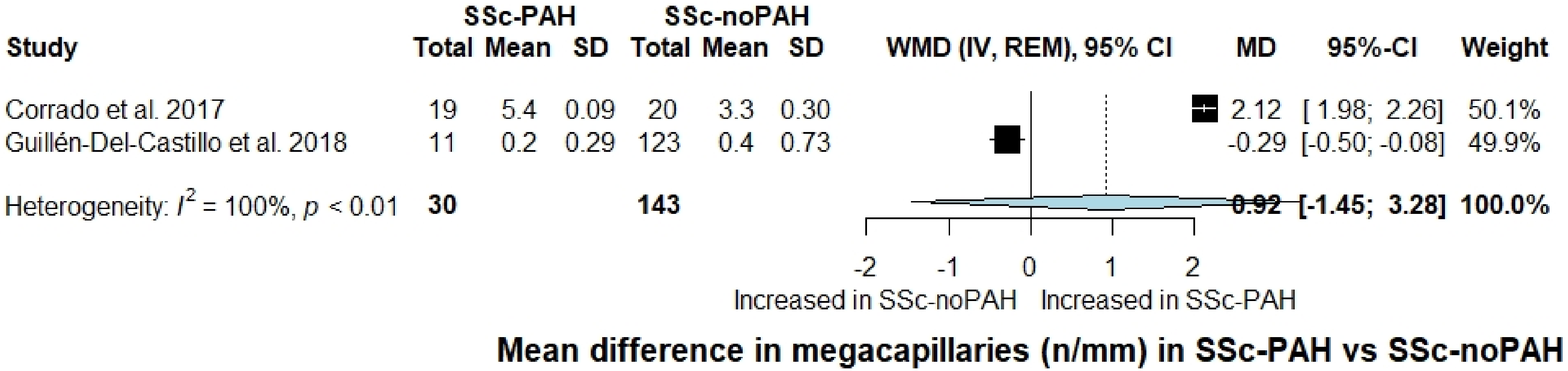

Figure 6.4: Forest plot of observational studies exploring megacapillaries (no/mm) in SSc-PAH versus SSc-noPAH patients. CI: confidence interval; IV: inverse variance; MD: mean difference; REM: Random Effects Model; SD: standard deviation; SSc-noPAH: Systemic sclerosis without Pulmonary arterial hypertension; SSc-PAH: Systemic sclerosis with Pulmonary arterial hypertension; WMD: weighted mean difference.

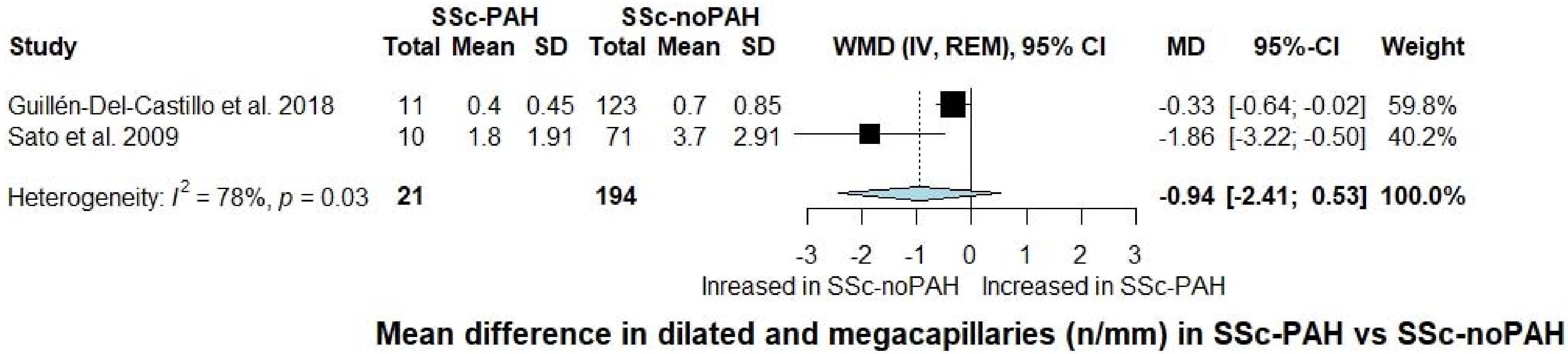

Figure 6.5: Forest plot of observational studies exploring the combination of dilated and mega- capillaries (no/mm) in SSc-PAH versus SSc-noPAH patients. CI: confidence interval; IV: inverse variance; MD: mean difference; REM: Random Effects Model; SD: standard deviation; SSc-noPAH: Systemic sclerosis without Pulmonary arterial hypertension; SSc-PAH: Systemic sclerosis with Pulmonary arterial hypertension; WMD: weighted mean difference.

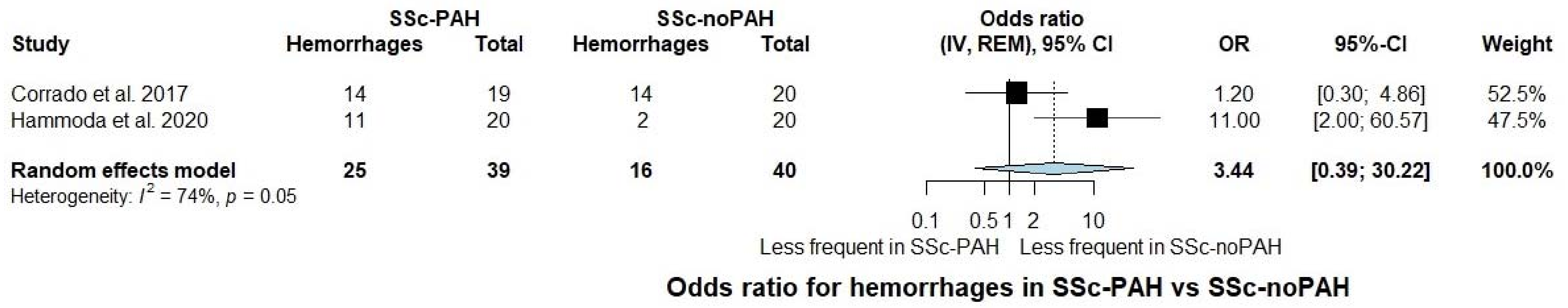

Figure 6.6: Forest plot of observational studies exploring occurrence of hemorrhages in SSc-PAH versus SSc-noPAH patients. CI: confidence interval; IV: inverse variance; MD: mean difference; REM: Random Effects Model; SD: standard deviation; SSc-noPAH: Systemic sclerosis without Pulmonary arterial hypertension; SSc-PAH: Systemic sclerosis with Pulmonary arterial hypertension; WMD: weighted mean difference.

Data Supplement 7: Forest plots of semi-quantitative parameters of nailfold capillaroscopy assessment

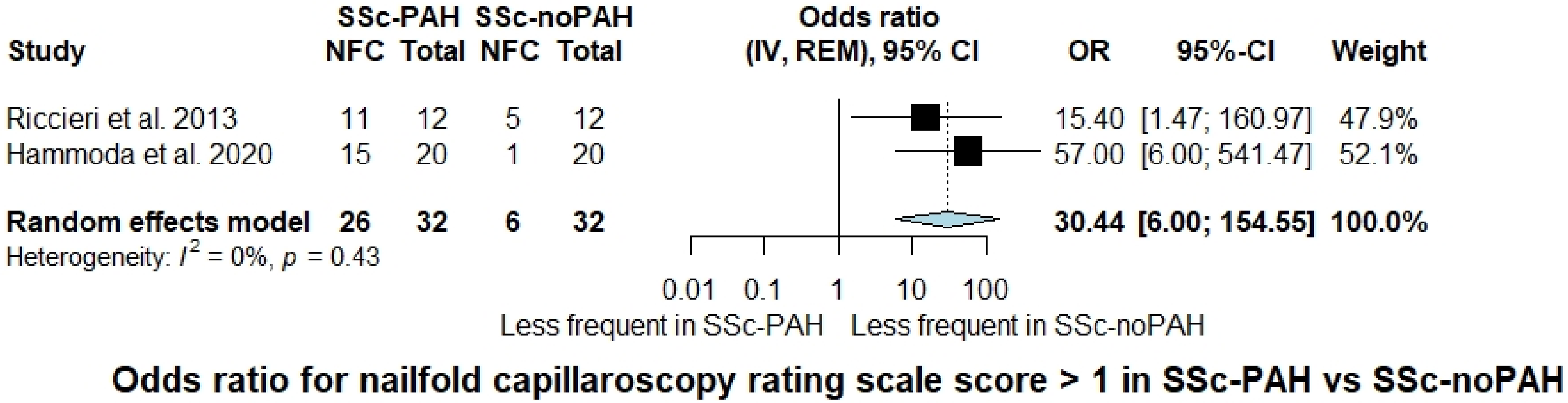

Figure 7.1: Forest plot of observational studies exploring occurrence of nailfold capillaroscopy rating scale score greater than 1 in SSc-PAH versus SSc-noPAH patients. CI: confidence interval; IV: inverse variance; MD: mean difference; NFC: Nailfold capillaroscopy rating scale score; REM: Random Effects Model; SD: standard deviation; SSc-noPAH: Systemic sclerosis without Pulmonary arterial hypertension; SSc-PAH: Systemic sclerosis with Pulmonary arterial hypertension; WMD: weighted mean difference.

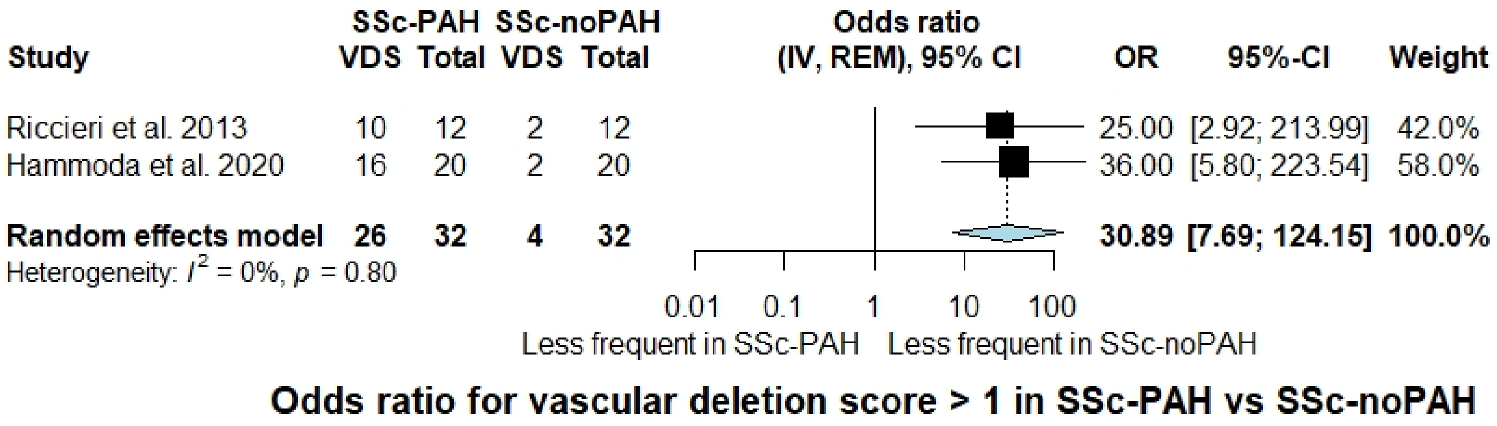

Figure 7.2: Forest plot of observational studies exploring occurrence of vascular deletion score greater than 1 in SSc-PAH versus SSc-noPAH patients. CI: confidence interval; IV: inverse variance; MD: mean difference; REM: Random Effects Model; SD: standard deviation; SSc-noPAH: Systemic sclerosis without Pulmonary arterial hypertension; SSc-PAH: Systemic sclerosis with Pulmonary arterial hypertension; VDS: Vascular deletion score; WMD: weighted mean difference.
